# Design of Multi-User Noncoherent Massive SIMO Systems for Scalable URLLC

**DOI:** 10.3390/e25091325

**Published:** 2023-09-12

**Authors:** Zheng Dong, He Chen, Jian-Kang Zhang

**Affiliations:** 1 School of Information Science and Engineering, Shandong University, Qingdao 266237, China; zhengdong@sdu.edu.cn; 2 Department of Information Engineering, The Chinese University of Hong Kong, Shatin, NT, Hong Kong SAR, China; 3 Department of Electrical and Computer Engineering, McMaster University, Hamilton, ON L8S 4K1, Canada

**Keywords:** scalable ultra-reliable low-latency communications (URLLC), massive SIMO, noncoherent communication, non-orthogonal multiple access (NOMA), uniquely decomposable constellation group

## Abstract

This paper develops and optimizes a non-orthogonal and noncoherent multi-user massive single-input multiple-output (SIMO) framework, with the objective of enabling scalable ultra-reliable low-latency communications (sURLLC) in Beyond-5G (B5G)/6G wireless communication systems. In this framework, the huge diversity gain associated with the large-scale antenna array in the massive SIMO system is leveraged to ensure ultra-high reliability. To reduce the overhead and latency induced by the channel estimation process, we advocate for the noncoherent communication technique, which does not need the knowledge of instantaneous channel state information (CSI) but only relies on large-scale fading coefficients for message decoding. To boost the scalability of noncoherent massive SIMO systems, we enable the non-orthogonal channel access of multiple users by devising a new differential modulation scheme to ensure that each transmitted signal matrix can be uniquely determined in the noise-free case and be reliably estimated in noisy cases when the antenna array size is scaled up. The key idea is to make the transmitted signals from multiple geographically separated users be superimposed properly over the air, such that when the sum signal is correctly detected, the signal sent by each individual user can be uniquely determined. To further enhance the average error performance when the array antenna number is large, we propose a max–min Kullback–Leibler (KL) divergence-based design by jointly optimizing the transmitted powers of all users and the sub-constellation assignments among them. The simulation results show that the proposed design significantly outperforms the existing max–min Euclidean distance-based counterpart in terms of error performance. Moreover, our proposed approach also has a better error performance compared to the conventional coherent zero-forcing (ZF) receiver with orthogonal channel training, particularly for cell-edge users.

## 1. Introduction

Driven by the relentless growth of wireless data traffic over the past four decades, modern wireless communication systems (from 2G to nowadays 5G) have been consistently engineered and developed, with the objective of providing better mobile broadband services, aiming to provide subscribers with ever-higher data rates. The trend is envisioned to continue in Beyond-5G (B5G)/6G [1,2].

Moreover, with the grand ambition of offering connectivity to anything that may benefit from being connected, 5G cellular systems support two new service categories in addition to the conventional mobile broadband one: massive machine-type communication (mMTC) [3] and ultra-reliable low-latency communication (URLLC) [4], which will still be key features for future 6G systems [5,6,7].

The mMTC service refers to providing wireless connectivity for a massive number (tens of thousands) of low-cost and low-energy machine-type devices (MTDs) in a relatively large area. The mMTC can find potential applications in smart metering, smart agriculture, logistics, fleet management, etc. The traffic of these applications is characterized as massive yet sporadic small-packet transmissions that require the support of high-spectrum efficiency and network scalability. Furthermore, the network maintenance cost can be huge due to the large amount of nodes. As such, ultra-high energy efficiency is expected to achieve long battery lifetimes in MTDs. On the other hand, URLLC is a service category that is not present in today’s mobile systems, which target mission-critical applications, requiring low end-to-end latency with high reliability. Examples include fault detection and isolation in power systems, detection and responses to hazardous road conditions, self-driving vehicles, remote surgery, smart factories, and augmented reality.

Nevertheless, with the continuing deployment of 5G cellular systems, in practice, it gradually becomes clear that the current 5G system cannot fulfill the promised vision of being an enabler for the “Internet of Everything”, especially the most innovative URLLC part, due to its inherent limitations [1]. While the enormous network capacity growth is achievable through conventional methods of moving to higher parts of the radio spectrum and network densification, realizing URLLC will involve a departure from the underlying theoretical principles of wireless communications. More specifically, the coupling and contradictory requirements of low latency and high reliability render the design of URLLC systems challenging, since wireless channels are highly dynamic and are susceptible to fading, interference, blockage, and high path losses, especially when there are many moving devices, metallic reflectors, and electromagnetic radiation equipment [8,9,10,11,12]. Such design challenges will be further escalated to provide the envisioned *scalable URLLC* (sURLLC) services in wireless systems beyond 5G. As defined in [1], sURLLC will scale the 5G URLLC across the device dimension by seamlessly integrating 5G URLLC with legacy mMTC. In sURLLC, the augmented triple reliability–latency–scalability trade-off will need to be carefully dealt with, which calls for a totally new design framework.

In wireless communications, channel diversity, which refers to a measure of transmitting multiple copies of the same information through independent links along different time/frequency/spatial axes, is one of the most important techniques for boosting system reliability by effectively combating channel fading and interference [13]. As the diversity order increases, wireless channels will gradually become more stable, and the chance of requesting retransmissions from the receiver side will correspondingly decrease [9]. However, in realizing sURLLC, time diversity is not preferred since achieving time diversity is at the cost of additional delay, especially in slow-fading channels. Moreover, delivering the information along distinct frequency channels will consume additional bandwidth, which is costly for operations under 6 GHz, where the frequency band has already been overcrowded [14]. Thus, harnessing the spatial diversity by deploying multiple antennas at the transmitter and/or receiver side is the most appealing solution, and there have been extensive studies on various diversity technologies for conventional MIMO systems; see, e.g., [15,16] and references therein.

Recently, massive multiple-input multiple-output (MIMO) technology [17], which scales up the conventional MIMO by deploying a large number of antennas at the transmitter and/or receiver side, is regarded as an indispensable building block for ensuring ultra-high reliability [9].  Massive MIMO has already been an integrating part of 5G communications due to its great potential [18,19]. The main advantages of massive MIMO include its high array gain, high spatial multiplexing gain, and immunity to fast fading in rich-scattering environments. The fluctuations of wireless channels can be averaged out in massive MIMO, and high reliability can be maintained for short packets without the need for strong channel coding. Despite the advantages mentioned above, the mechanisms of leveraging massive MIMO for realizing sURLLC are still largely unexplored.

The reliability and latency gains associated with massive MIMO systems critically depend on the acquisition of the instantaneous channel state information (CSI) [20]. In conventional communication systems, the estimation of instantaneous CSI is commonly achieved by transmitting certain known pilot symbols that are orthogonal to different users, where the channel estimation overhead is relatively low compared with the long data payload. However, the packets in sURLLC applications are typically very short and, thus, the overhead induced by channel estimation becomes non-negligible and will reduce the effective transmission rate significantly [8]. Moreover, sending pilots will cause significant delays in short-packet communications, especially over fast-fading channels where the pilot symbols need to be dense in the time-frequency grid. As a matter of fact, obtaining instantaneous CSI is one of the most severe limiting factors to exploiting the full potential of massive MIMO, where the latency introduced by the channel estimation in massive MIMO constitutes a major barrier to meeting the extreme delay requirement [9]. To reduce latency in massive MIMO, the transmission protocol should rely as little as possible on the channel knowledge of small-scale fading [9]. Nevertheless, the knowledge of channel statistics remains crucial to provide high-reliability requirements, especially when the precise knowledge of instantaneous CSI is not available. Noncoherent detection, where no instantaneous CSI is required, can be the key supporting asset for low-latency applications. It was shown in [21] that noncoherent transmission is more energy-efficient than pilot-assisted transmission schemes, even when the number of pilot symbols and their power are optimized.

We note that there have been considerable efforts in designing single-user noncoherent massive single-input multiple-output (SIMO) systems, see e.g., [22,23,24,25,26,27,28], which demonstrate that a simple energy-based modulation and detector can be sufficient for reliable detection by leveraging the massive number of antennas. By considering the single-user scenario, these works implicitly assume that an orthogonal multiple access (OMA) mechanism (e.g., time-division multiple access) has been adopted at the data link layer to support the co-existence of multiple users. However, OMA mechanisms normally have poor scalability—the channel access latency scales up linearly as the number of end-devices increases, and, thus, are no longer suitable for the more challenging sURLLC applications with a large number of forecasted devices. One effective solution to address this scalability issue is to break the orthogonality of existing OMA protocols and empower a new non-orthogonal and noncoherent massive MIMO (nn-mMIMO) framework. It is worth mentioning here that non-orthogonal multiple access (NOMA) has recently received tremendous attention from the mobile communication research community as a promising technology for 5G cellular system, see a recent comprehensive survey [29], where the primary goal of applying NOMA is to boost spectral efficiency and user fairness. Existing NOMA solutions along this research line normally require the estimation of instantaneous CSI, such that the optimization of power allocation/control for different signal streams can be conducted at the transmitter side and successive interference cancellation can be implemented for detecting multiple user signals at the receiver side. These NOMA solutions are, thus, not applicable anymore for nn-mMIMO-enabled sURLLC applications.

Enabling NOMA in massive MIMO is straightforward when the instantaneous CSI is available, which can be achieved by applying spatial division multiple access (SDMA). However, how to support the non-orthogonal access of multiple users at the same time in noncoherent massive MIMO systems is a non-trivial task as beamforming techniques cannot be used anymore. Very recently, a new constellation domain-based NOMA methodology, with the objective of enabling nn-mMIMO, was developed in [30,31,32,33], which allows the simultaneous channel access of multiple devices at the data link layer without the availability of instantaneous CSI at the physical layer. However, all the designs in [30,31,32,33] only considered one-shot communications (i.e., the received signal is decoded in a symbol-by-symbol manner). As such, the phase information of the transmitted signals is lost at the receiver side and, thus, only unipolar PAM constellations can be used, which largely limits the system reliability performance as the number of devices increases.

In regard to enabling sURLLC, in this paper, we develop a new nn-mMIMO framework that can perform joint noncoherent detection of the uplink signals from multiple devices over more than one time slot, where the transmitted signals are allowed to use the more robust QAM constellations. The main contributions of this paper are two-fold:

Firstly, we apply a noncoherent maximum likelihood (ML) receiver, which relies only on the second-order channel statistics, and no instantaneous CSI is needed at either the transmitter or receiver sides. For the considered ML receiver, we systematically design a uniquely factorable multi-user space-time modulation (UF-MUSTM) scheme to enable the concurrent transmission of multiple devices to a noncoherent receiver equipped with a large number of antennas. We further identify the necessary and sufficient conditions for the receiver to recover the transmitted signals from all users. Note that our design connects to the conventional space-time code design. To date, most of the existing space-time code designs, such as [16,34,35,36], considered point-to-point MIMO systems, where all the transmitting antennas are connected to the same transmitter; hence, the transmitted information-carrying signals are accessible by all the antennas. However, in our considered UF-MUSTM-based nn-mMIMO system, the signals transmitted from different users are not allowed to fully collaborate, which dramatically limits the codebook design. Particularly, the widely used unitary space-time code design is, in general, intractable for the considered multi-user massive MIMO system.

Secondly, we further optimize the proposed design framework by jointly designing the constellations of multiple users. We note that the performance analysis for the non-unitary codeword of MUSTM is extremely challenging, if not possible, as shown in [34,35]. Confronting such a challenge, we propose a max–min Kullback–Leibler (KL) divergence-based design criterion, where we jointly optimize the transmit powers of all users and the sub-constellation assignments among them. Note that the basic idea of this paper was presented in the conference version [37], where we only consider the simple scenario where all users adopt 4-QAM. In this paper, we expand the design to a more general scenario where all users can utilize larger QAM, not necessarily of the same orders. This introduces added complexity to the optimization problem, making it more challenging to solve. We manage to resolve the formulated optimization problem in closed form. Simulations are provided to demonstrate the superiority of the proposed design over the benchmarking schemes.

The remainder of this paper is organized as follows. In Section 2, we describe the system model, the noncoherent detector, as well as the signal design. The design and optimization of the proposed UF-MUSTM framework are elaborated in Section 3. Simulations are conducted and the corresponding results are discussed in Section 4. The conclusions are drawn in Section 5.

## 2. System Model, Noncoherent Detector, and Signal Design

### 2.1. System Model and Noncoherent ML Detector

We consider a massive MIMO system, consisting of *K* single-antenna users transmitting simultaneously to a base station (BS) with *M* (M≫K) receiving antennas on the same time-frequency grid. By using a discrete-time complex baseband-equivalent model, the received signal at the antenna array of BS in the *t*-th time slot (each time slot refers to one symbol duration throughout this paper), is defined as $y_{t}={\left[y_{1,t},\ldots ,y_{M,t}\right]}^{T}$, can be expressed by

(1) $$\begin{array}{c} y_{t}=Hx_{t}+\xi_{t}, \end{array}$$

where $x_{t}={\left[x_{1,t},\ldots ,x_{K,t}\right]}^{T}$ represents the transmitted signals from all *K* users, $\xi_{t}$ is an additive circularly symmetric complex Gaussian (CSCG) noise vector with covariance $\sigma^{2}I_{M}$. We let $H=GD^{1/2}$ denote the M×K complex channel matrix between the receiver antenna array and all users, where G characterizes the small-scale fading caused by local scattering while $D=\mathrm{diag}\{\beta_{1},\ldots ,\beta_{K}\}$ with $\beta_{k}>0$ capturing the propagation loss due to distance and shadowing. All the entries of G are assumed to be i.i.d. complex Gaussian distributed with zero mean and unit variance. The channel coefficients are assumed to suffer from block fading, which are quasi-static in the current block and change to other independent values in the next block with a channel coherence time $T_{c}\geq K$. We consider a space-time block modulation (STBM) [35] scheme over *T* time slots and the received signal vectors can be stacked together into a matrix form given by

(2) $$\begin{array}{c} Y_{T}=HX_{T}+\Xi_{T}, \end{array}$$

where $Y_{T}=\left[y_{1},\ldots ,y_{T}\right]$, $X_{T}=\left[x_{1},\ldots ,x_{T}\right]$, and $\Xi_{T}=\left[\xi_{1},\cdots ,\xi_{T}\right]$.

**Assumption** **1.** *Throughout this paper, we adopt the following assumptions:*

*1*. *The small-scale channel fading matrix G is completely unknown to the BS and all the users, while the large-scale fading matrix D is available at the BS, which will be leveraged to optimize the system performance;*
*2*. *The transmitted signals are subject to an instantaneous average power constraint (note that our design can be directly extended to the case with the peak power constraint): $E\{|x_{k,t}{|}^{2}\}\leq P_{k}$, k=1,…,K, t=1,…,T. For convenience, we assume that the users are labeled in ascending order with $P_{1}\beta_{1}\leq \ldots \leq P_{K}\beta_{K}$.*

In this work, we apply a noncoherent ML detector, which is optimal for uniformly distributed discrete input signals in terms of error probability. We note that (2) can be reformulated as $Y_{T}^{H}=X_{T}^{H}D^{1/2}G^{H}+\Xi_{T}^{H}$. With the help of [38], the vectorized form of the received signal can then be written as

$$\begin{array}{c} y=\mathrm{vec}\left(Y_{T}^{H}\right)=\left(I_{M}⊗X_{T}^{H}D^{1/2}\right)\mathrm{vec}\left(G^{H}\right)+\mathrm{vec}\left(\Xi_{T}^{H}\right). \end{array}$$

As all the entries of G and Ξ are i.i.d. CSCG, we immediately have E[y]=0, and the covariance matrix of y can be calculated as $R_{y|X_{T}}=E\left[yy^{H}\right]=I_{M}⊗\left(X_{T}^{H}DX_{T}+\sigma^{2}I_{T}\right)$. The conditional distribution of the received signal y at BS for any transmitted signal matrix $X_{T}$ can then be given by $p\left(y|X_{T}\right)=\frac{1}{\pi^{KM}\det\left(R_{y|X_{T}}\right)}\exp\left(-y^{H}R_{y|X_{T}}^{-1}y\right)$, where $R_{y|X_{T}}=I⊗\left(X_{T}^{H}DX_{T}+\sigma^{2}I_{T}\right)$. The noncoherent ML detector can estimate the transmitted information-carrying matrix from the received signal vector y by resolving the following optimization problem:

(3) $$\begin{array}{c} {\hat{X}}_{T}=\arg\min_{X_{T}}\;y^{H}R_{y|X_{T}}^{-1}y+\log\det\left(R_{y|X_{T}}\right). \end{array}$$

From (3), we can observe that the detector relies on the sufficient statistics of the transmitted signal matrix $R_{y|X_{T}}=I⊗\left(X_{T}^{H}DX_{T}+\sigma^{2}I_{T}\right)$. The detailed discussion regarding the signal design is given in the following subsection.

### 2.2. Unique Identification of the Transmitted Signal Matrix

In this subsection, we first identify what conditions the transmitted signal matrix must satisfy to ensure the unique identification of the transmitted signal matrix $X_{T}$. We can observe from (3) that, to achieve reliable communication between all users and the BS in the considered nn-mMIMO system, the BS must be able to uniquely determine each transmitted signal matrix $X_{T}$ once $R=X_{T}^{H}DX_{T}$ is identified, which can be formally stated as follows:

**Proposition** **1.** *For the multi-user nn-mMIMO system described in* (2) *reliable communications necessitate the following condition for the transmitted signal matrix selected from $M^{K\times T}\subseteq C^{K\times T}$. If and only if there exist any two signal matrices $X_{T},{\tilde{X}}_{T}\in M^{K\times T}$ satisfying $X_{T}^{H}DX_{T}={\tilde{X}}_{T}^{H}D{\tilde{X}}_{T}$, then we have $X_{T}={\tilde{X}}_{T}$.*

The proof is provided in Appendix A.1. Inspired by Proposition 1, to facilitate our system design, we introduce the concept of uniquely factorable multi-user space-time modulation (UF-MUSTM), the formal definition of which is given as follows:

**Definition** **1.** *A multi-user space-time modulation codebook $S^{K\times T}\subseteq C^{K\times T}$ is said to form a UF-MUSTM codebook if for any pair of codewords $S,\tilde{S}\in S^{K\times T}$ satisfying $S^{H}S={\tilde{S}}^{H}\tilde{S}$, we have $S=\tilde{S}$.*

Definition 1 motivates us to design a UF-MUSTM codebook for the considered nn-mMIMO system. Therefore, our primary task in the rest of this paper is to develop a new framework for a systematic design of such a UF-MUSTM $S^{K\times T}$.

Before proceeding, it is worth clarifying that the UF-MUSTM code design is fundamentally different from the existing noncoherent space-time code/modulation designs. Specifically,

- For the considered UF-MUSTM-based nn-mMIMO system, the signals transmitted from different users cannot fully collaborate; hence, the widely used unitary space-time code design is intractable for the considered system. This is fundamentally different from most conventional space-time code designs for the point-to-point MIMO system, where all transmitting antennas are connected to the same transmitter [16,35]. Note that the error performance analysis of the non-unitary codeword of MUSTM is very challenging, as shown in [34].
- Our design is asymptotically optimal when the number of BS antennas goes to infinity while keeping the transmitted power fixed. This is in contrast to most previous space-time coding designs, which considered the asymptotic regime with the signal-to-noise ratio (SNR) going to infinity [34,35,36].

## 3. Design and Optimization of UF-MUSTM Framework

In this section, we present a UF-MUSTM framework with a slot-by-slot noncoherent ML detector. We find that when the number of receiving antennas increases, the pairwise error probability (PEP) between two codewords will be dominated by the KL divergence between them. Motivated by this fact, a max–min KL divergence design criterion is proposed to optimize the transmit powers of all users and the sub-constellations assignment among them.

### 3.1. KL Divergence between Transmitted Space-Time Modulation Codewords

In practice, the computational complexity of the optimal noncoherent ML detector described in (3) could be prohibitively high. Furthermore, the error performance analysis results available for the block transmission with general block size and ML receiver are too complicated to reveal insightful results for the input codeword design and the corresponding power allocation [34]. To resolve these problems and reduce the receiver complexity, our main objective is to input a small block size into the ML receiver. If only one time slot is involved in the ML detector given in (3), i.e., when T=1, the correlation matrix $R=X_{T}^{H}DX_{T}$ degenerates into a real scalar $x_{1}^{H}Dx_{1}=\sum_{k=1}^{K}\beta_{k}{\left|x_{k,1}\right|}^{2}$, where the phase information of the transmitted symbols is lost and information bits from all users can only be modulated on the amplitudes of the transmitted symbols. Such a design typically has a low spectral efficiency [22,23,30]. To improve the spectrum efficiency by allowing constellation with phase information being transmitted by all users, we need to feed the signals received in at least two time slots into the ML decoder [34,35,39].

As an initial attempt, in this paper, we focus on a slot-by-slot ML detection over the first and *t*-th time slots, which is similar to the differential modulation with hard-decision-based noncoherent multi-user detection. More specifically, we let the transmitted signal matrix be $X_{T}=\left[x_{1},\ldots ,x_{T}\right]$. For detection purposes, we now stack the transmitted signal of the first and the *t*-th time slot as $X_{t}=\left[x_{1},x_{t}\right]$, and then make the decision on $Y_{t}=\left[y_{1},y_{t}\right]$ by using (3). For simplicity, we consider the transmitted signal from the first and second time slots, i.e., $X_{2}=\left[x_{1},x_{2}\right]$, hereafter, and the case of $X_{t}$ follows similarly. We denote $R_{y|X_{2}}=I⊗R_{2}$, in which

(4) $$\begin{array}{cc} \; & R_{2}=X_{2}^{H}DX_{2}+\sigma^{2}I_{2}=\left[\begin{array}{cc} x_{1}^{H}Dx_{1}+\sigma^{2} & x_{1}^{H}Dx_{2}\\ x_{2}^{H}Dx_{1} & x_{2}^{H}Dx_{2}+\sigma^{2} \end{array}\right]. \end{array}$$

By (4), we have

(5) $$\begin{array}{c} R_{2}^{-1}=\frac{1}{\left(x_{1}^{H}Dx_{1}+\sigma^{2}\right)\left(x_{2}^{H}Dx_{2}+\sigma^{2}\right)-{\left|x_{1}^{H}Dx_{2}\right|}^{2}}\left[\begin{array}{cc} x_{2}^{H}Dx_{2}+\sigma^{2} & -x_{1}^{H}Dx_{2}\\ -x_{2}^{H}Dx_{1} & x_{1}^{H}Dx_{1}+\sigma^{2} \end{array}\right]. \end{array}$$

As a consequence, the ML receiver can be reformulated as follows

(6) $$\begin{array}{cc} {\hat{X}}_{2} & ={\arg\min}_{X_{2}}\;y^{H}R_{y|X_{2}}^{-1}y+\log\det\left(R_{y|X_{2}}\right)\\ \; & ={\arg\min}_{X_{2}}\;\frac{\left(x_{1}^{H}Dx_{1}+\sigma^{2}\right){∥y_{2}∥}^{2}+\left(x_{2}^{H}Dx_{2}+\sigma^{2}\right){∥y_{1}∥}^{2}-2ℜ\left(x_{1}^{H}Dx_{2}y_{2}^{H}y_{1}\right)}{\left(x_{1}^{H}Dx_{1}+\sigma^{2}\right)\left(x_{2}^{H}Dx_{2}+\sigma^{2}\right)-{\left|x_{1}^{H}Dx_{2}\right|}^{2}}\\ \; & \;\;\;\;\;+M\ln\left[\left(x_{1}^{H}Dx_{1}+\sigma^{2}\right)\left(x_{2}^{H}Dx_{2}+\sigma^{2}\right)-{\left|x_{1}^{H}Dx_{2}\right|}^{2}\right], \end{array}$$

where $y_{1}$ and $y_{2}$ are the received signal vectors in the first and second time slots, respectively. It can be observed that the diagonal entries in (4) are $x_{1}^{H}Dx_{1}=\sum_{k=1}^{K}\beta_{k}{\left|x_{k,1}\right|}^{2}$ and $x_{2}^{H}Dx_{2}=\sum_{k=1}^{K}\beta_{k}{\left|x_{k,2}\right|}^{2}$, in which the phase information is lost, while the off-diagonal term is $x_{1}^{H}Dx_{2}=\sum_{k=1}^{K}\beta_{k}x_{k,1}^{*}x_{k,2}=\sum_{k=1}^{K}\beta_{k}|x_{k,1}\left|\right|x_{k,2}|\exp\left(j\arg\left(x_{k,2}\right)-j\arg\left(x_{k,1}\right)\right)$, indicating that we can transmit a known reference signal vector $x_{1}$ in the first time slot and then transmit the information-bearing signal vector $x_{2}$ to imitate a “differential-like” transmission [40]. The exact PEP is extremely hard to evaluate for the matrix $X_{2}$ given above. Moreover, the exact expression for the PEP does not seem to be tractable for further optimization. Inspired by the Chernoff–Stein Lemma, when the number of receiver antennas *M* goes to infinity, the PEP will go to zero, exponentially, where the exponent is determined by the KL divergence [35]. Hence, we propose using the KL divergence between the conditional distributions of the received signals for different inputs as the design criterion, thanks to its mathematical tractability.

We now derive the KL divergence between the received signals induced by the transmitted signals matrices $X_{2}=\left[x_{1},x_{2}\right]$ and ${\tilde{X}}_{2}=\left[{\tilde{x}}_{1},{\tilde{x}}_{2}\right]$, which is also the expectation of the likelihood function between two received signal vectors. Essentially, the likelihood function between the received signal vectors corresponding to the two transmitted signals converge in probability to the KL divergence as the number of receiver antennas increases [35]. More specifically, the KL divergence between the received signals corresponding to the transmitted matrix $X_{2}$ and ${\tilde{X}}_{2}$ can be calculated as

$$\begin{array}{cc} \; & D_{\mathrm{KL}}^{\left(M\right)}(X_{2}\left|\right|{\tilde{X}}_{2})=E_{f(y|X_{2})}\left\{\ln\left(\frac{f(y|{\tilde{X}}_{2})}{f(y|X_{2})}\right)\right\}\\ \; & =E_{f(y|X_{2})}\left\{\ln\left(\frac{\det(R_{y|X_{2}})}{\det(R_{y|{\tilde{X}}_{2}})}\right)+\left(y^{H}R_{y|{\tilde{X}}_{2}}^{-1}y-y^{H}R_{y|X_{2}}^{-1}y\right)\right\}\\ \; & =E_{f(y|X_{2})}\left\{\mathrm{tr}\left((R_{y|{\tilde{X}}_{2}}^{-1}-R_{y|X_{2}}^{-1})yy^{H}\right)\right\}+\ln\left(\frac{\det(R_{y|X_{2}})}{\det(R_{y|{\tilde{X}}_{2}})}\right)\\ \; & =\mathrm{tr}\left[\left(R_{y|{\tilde{X}}_{2}}^{-1}-R_{y|X_{2}}^{-1}\right)R_{y|X_{2}}\right]+\ln\left(\frac{\det(R_{y|X_{2}})}{\det(R_{y|{\tilde{X}}_{2}})}\right)\\ \; & =M\;D_{\mathrm{KL}}(X_{2}\left|\right|{\tilde{X}}_{2}), \end{array}$$

in which

(7) $$\begin{array}{cc} D_{\mathrm{KL}}(X_{2}\left|\right|{\tilde{X}}_{2}) & =\mathrm{tr}\left[\left(X_{2}^{H}DX_{2}+\sigma^{2}I_{2}\right){\left({\tilde{X}}_{2}^{H}D{\tilde{X}}_{2}+\sigma^{2}I_{2}\right)}^{-1}\right]\\ \; & \;-\ln\left\{\det[\left(X_{2}^{H}DX_{2}+\sigma^{2}I_{2}\right){\left({\tilde{X}}_{2}^{H}D{\tilde{X}}_{2}+\sigma^{2}I_{2}\right)}^{-1}]\right\}-2. \end{array}$$

We can observe from the above expression that $D_{\mathrm{KL}}(X_{2}\left|\right|{\tilde{X}}_{2})$ is actually the KL divergence when there is only one receiving antenna. Due to the assumption of the independence of channel coefficients, and the KL divergence with *M* antennas $D_{\mathrm{KL}}^{\left(M\right)}(X_{2}\left|\right|{\tilde{X}}_{2})$ is *M* times $D_{\mathrm{KL}}(X_{2}\left|\right|{\tilde{X}}_{2})$.

### 3.2. QAM Division-Based Multi-User Space-Time Modulation

The main objective of this subsection is to develop a new QAM division-based MUSTM design framework for the considered nn-mMIMO system. The design is built upon the uniquely decomposable constellation group (UDCG) originally proposed in [41,42] for the commonly used spectrally efficient QAM signaling. We now introduce the definition of UDCG as follows:

**Definition** **2.** *A group of constellations ${\left\{X_{k}\right\}}_{k=1}^{K}$ form a UDCG, denoted by $\left\{\sum_{k=1}^{K}x_{k}:x_{k}\in X_{k}\right\}={⊎}_{k=1}^{K}X_{k}=X_{1}⊎\ldots ⊎X_{K}$, if there exist two groups of $x_{k},{\tilde{x}}_{k}\in X_{k}$ for k=1,⋯,K such that $\sum_{k=1}^{K}x_{k}=\sum_{k=1}^{K}{\tilde{x}}_{k}$, then we have $x_{k}={\tilde{x}}_{k}$ for k=1,⋯,K. *

As PAM and QAM constellations are commonly used in modern digital communications, which have simple geometric structures, we now provide the following construction of UDCG.

**Lemma** **1.** *The UDCG with PAM and QAM constellations can be constructed as follows:*

*(1)* UDCG with PAM constellation*: For two given positive integers, K and N (N≥K), and a nonnegative integer sequence, ${\left\{N_{k}\right\}}_{k=1}^{K}$, satisfying $\sum_{k=1}^{K}N_{k}=N$, a $2^{N}$-ary PAM constellation $G=\{\pm \left(m-\frac{1}{2}\right)\Delta :m=1,\ldots ,2^{N-1}\}$, with *Δ* being the minimum Euclidean distance between the constellation points, can be uniquely decomposed into the sum of K sub-constellations ${\left\{X_{k}\right\}}_{k=1}^{K}$ denoted by $G={⊎}_{k=1}^{K}X_{k}$, where $X_{1}={\left\{\pm (m-\frac{1}{2})\Delta \right\}}_{m=1}^{2^{N_{1}-1}}$, and $X_{k}={\left\{\pm \left(m-\frac{1}{2}\right)\times 2^{\sum_{\ell =1}^{k-1}N_{\ell }}\Delta \right\}}_{m=1}^{2^{N_{k}-1}}$ for k≥2.*

*(2)* UDCG with QAM constellation*: For two positive integers K and $N=N_{I}+N_{Q}$ (N≥K), with $N_{I}$ and $N_{Q}$ being nonnegative integers that denote the sizes of the in-phase and quadrature components, respectively. Let ${\left\{N_{I,k}\right\}}_{k=1}^{K}$ and ${\left\{N_{Q,k}\right\}}_{k=1}^{K}$ denote two given nonnegative integer sequences satisfying $N_{I}=\sum_{k=1}^{K}N_{I,k}$ and $N_{Q}=\sum_{k=1}^{K}N_{Q,k}$ with $N_{k}=N_{I,k}+N_{Q,k}>0$. Then, there exists a PAM and QAM mixed constellation $Q={⊎}_{k=1}^{K}X_{k}$ such that $X_{k}=X_{I,k}⊎jX_{Q,k}$, with $jX_{Q,k}=\left\{jx:x\in X_{Q,k}\right\}$, where $Q_{I}={⊎}_{k=1}^{K}X_{I,k}$ and $Q_{Q}={⊎}_{k=1}^{K}X_{Q,k}$ are two PAM UDCGs according to the rate allocation ${\left\{N_{I,k}\right\}}_{k=1}^{K}$ and ${\left\{N_{Q,k}\right\}}_{k=1}^{K}$, respectively.  *

With the concept of UDCG, we are now ready to propose a QAM division-based UF-MUSTM for the considered nn-mMIMO system with a noncoherent ML receiver given in (6). The structure of each transmitted signal matrix is given by $X_{2}=\left[x_{1},x_{2}\right]=D^{-1/2}\Pi S_{2}$, in which

(8) $$\begin{array}{cc} S_{2} & =\left[s_{1},s_{2}\right]=\left[\begin{array}{cc} \frac{1}{\sqrt{p_{1}}} & \sqrt{p_{1}}s_{1}\\ \frac{1}{\sqrt{p_{2}}} & \sqrt{p_{2}}s_{2}\\ \vdots & \vdots \\ \frac{1}{\sqrt{p_{K}}} & \sqrt{p_{K}}s_{K} \end{array}\right]. \end{array}$$

In our design, the diagonal matrix $D^{-1/2}$ is used to compensate for the different large-scale fading among various users. The vector $p=[p_{1},\ldots ,p_{K}]$ is introduced to adjust the relative transmitting powers between all users, and $s=[s_{1},\ldots ,s_{K}]$ is the information-carrying vector. The instantaneous power constraint can be given by $E\{|x_{k,t}{|}^{2}\}\leq P_{k}$, k=1,…,K and t=1,2. We let $s_{k}\in X_{k}$, where all $X_{k}$’s constitute a UDCG with sum-QAM constellation Q, such that $Q={⊎}_{k=1}^{K}X_{k}$ as defined in Lemma 1. The rate allocation between the *K* users is based on the sum-decomposition, such that $\sum_{k=1}^{K}N_{k}=N$, in which $N_{k}=N_{I,k}+N_{Q,k}=\log_{2}\left(\right|X_{k}\left|\right)$ denotes the bit rate of the user constellation $X_{k}$. The matrix

(9) $$\begin{array}{c} \Pi ={\left[e_{\pi \left(1\right)},\ldots ,e_{\pi (K)}\right]}^{T} \end{array}$$

is a permutation matrix, where $e_{k}$ denotes a standard basis column vector of length *K* with 1 in the *k*-th position and 0 in other positions. π:{1,…,K}→{1,…,K} is a permutation over *K* elements characterized by $\left(\begin{array}{cccc} 1 & 2 & \ldots & K\\ \pi (1) & \pi (2) & \ldots & \pi (K) \end{array}\right)$. We also let $\pi^{-1}:\left\{1,\ldots ,K\right\}\to \left\{1,\ldots ,K\right\}$ be a permutation such that $\pi^{-1}\left(\pi \left(k\right)\right)=k$ for k=1,…,K. From the above definition, we immediately have $\Pi^{T}\Pi =I_{K}$.

For the transmitted signal matrix $X_{2}$, we have the following desired properties:

**Proposition** **2.** *Consider $X_{2}=D^{-1/2}\Pi S_{2}$ and ${\tilde{X}}_{2}=D^{-1/2}\Pi {\tilde{S}}_{2}$, where $S_{2}$ and ${\tilde{S}}_{2}$ belong to $S^{K\times 2}$ as described in Definition 1. If $X_{2}^{H}DX_{2}={\tilde{X}}_{2}^{H}D{\tilde{X}}_{2}$, then we have $X_{2}={\tilde{X}}_{2}$.*

The proof of Proposition 2 is given in Appendix A.2.

### 3.3. User-Constellation Assignment and Power Allocation

To further enhance the system reliability performance, we now optimize the user-constellation assignment policy π and power allocation vector p for the proposed nn-mMIMO framework. For the transmitted signal matrix considered in (8), we have

(10) $$\begin{array}{cc} \; & X_{2}^{H}DX_{2}+\sigma^{2}I_{2}=\left[\begin{array}{cc} s_{1}^{H}s_{1}+\sigma^{2} & s_{1}^{H}s_{2}\\ s_{2}^{H}s_{1} & s_{2}^{H}s_{2}+\sigma^{2} \end{array}\right]=\left[\begin{array}{cc} \sum_{k=1}^{K}1/p_{k}+\sigma^{2} & \sum_{k=1}^{K}s_{k}\\ \sum_{k=1}^{K}s_{k}^{*} & \sum_{k=1}^{K}p_{k}{\left|s_{k}\right|}^{2}+\sigma^{2} \end{array}\right],\\ \; & {\tilde{X}}_{2}^{H}D{\tilde{X}}_{2}+\sigma^{2}I_{2}=\left[\begin{array}{cc} s_{1}^{H}s_{1}+\sigma^{2} & s_{1}^{H}{\tilde{s}}_{2}\\ {\tilde{s}}_{2}^{H}s_{1} & {\tilde{s}}_{2}^{H}{\tilde{s}}_{2}+\sigma^{2} \end{array}\right]=\left[\begin{array}{cc} \sum_{k=1}^{K}1/p_{k}+\sigma^{2} & \sum_{k=1}^{K}{\tilde{s}}_{k}\\ \sum_{k=1}^{K}{\tilde{s}}_{k}^{*} & \sum_{k=1}^{K}p_{k}{\left|{\tilde{s}}_{k}\right|}^{2}+\sigma^{2} \end{array}\right]. \end{array}$$

We can see from (10) that $X_{2}^{H}DX_{2}+\sigma^{2}I_{2}$ and ${\tilde{X}}_{2}^{H}D{\tilde{X}}_{2}+\sigma^{2}I_{2}$ are independent of the permutation function π, but depends on the power allocation vector $p={\left[p_{1},\ldots ,p_{K}\right]}^{T}$, and the information carrying vectors $s={\left[s_{1},\ldots ,s_{K}\right]}^{T}$ and $\tilde{s}={\left[{\tilde{s}}_{1},\ldots ,{\tilde{s}}_{K}\right]}^{T}$. In this case, the ML receiver given in (6) can be further simplified as

(11) $$\begin{array}{cc} {\hat{X}}_{2} & =\underset{X_{2}}{\arg\min}\;\frac{a{∥y_{2}∥}^{2}+b{∥y_{1}∥}^{2}-2ℜ\left(cy_{2}^{H}y_{1}\right)}{ab-{\left|c\right|}^{2}}+M\ln\left(ab-{\left|c\right|}^{2}\right), \end{array}$$

in which $a=\sum_{k=1}^{K}1/p_{k}+\sigma^{2}$, $b=\sum_{k=1}^{K}p_{k}{\left|s_{k}\right|}^{2}+\sigma^{2}$, and $c=\sum_{k=1}^{K}s_{k}$. Inserting (10) into (7), and after some algebraic manipulations, we have

$$\begin{array}{cc} \; & D_{\mathrm{KL}}(X_{2}\left|\right|{\tilde{X}}_{2})=f_{1}\left(p,s,\tilde{s}\right)+f_{2}\left(p,s,\tilde{s}\right), \end{array}$$

where

$$\begin{array}{cc} \; & f_{1}\left(p,s,\tilde{s}\right)=\frac{\left(\sum_{k=1}^{K}\frac{1}{p_{k}}+\sigma^{2}\right)\left(\sum_{k=1}^{K}p_{k}{\left|s_{k}\right|}^{2}+\sigma^{2}\right)-|\sum_{k=1}^{K}s_{k}{|}^{2}}{\left(\sum_{k=1}^{K}\frac{1}{p_{k}}+\sigma^{2}\right)\left(\sum_{k=1}^{K}p_{k}{\left|{\tilde{s}}_{k}\right|}^{2}+\sigma^{2}\right)-|\sum_{k=1}^{K}{\tilde{s}}_{k}{|}^{2}}\\ \; & \;\;\;-\ln\left[\frac{\left(\sum_{k=1}^{K}\frac{1}{p_{k}}+\sigma^{2}\right)\left(\sum_{k=1}^{K}p_{k}{\left|s_{k}\right|}^{2}+\sigma^{2}\right)-|\sum_{k=1}^{K}s_{k}{|}^{2}}{\left(\sum_{k=1}^{K}\frac{1}{p_{k}}+\sigma^{2}\right)\left(\sum_{k=1}^{K}p_{k}{\left|{\tilde{s}}_{k}\right|}^{2}+\sigma^{2}\right)-|\sum_{k=1}^{K}{\tilde{s}}_{k}{|}^{2}}\right]-1,\\ \; & f_{2}\left(p,s,\tilde{s}\right)=\frac{|\sum_{k=1}^{K}s_{k}-\sum_{k=1}^{K}{\tilde{s}}_{k}{|}^{2}}{\left(\sum_{k=1}^{K}\frac{1}{p_{k}}+\sigma^{2}\right)\left(\sum_{k=1}^{K}p_{k}{\left|{\tilde{s}}_{k}\right|}^{2}+\sigma^{2}\right)-|\sum_{k=1}^{K}{\tilde{s}}_{k}{|}^{2}}. \end{array}$$

Recall that the power constraints are $E\{|x_{k,t}{|}^{2}\}\leq P_{k}$ for k=1,…,K and t=1,2. That is, for the first and second time slots, we have $E\{|x_{k,1}{|}^{2}\}=\frac{1}{p_{\pi (k)}\beta_{k}}\leq P_{k}$, and $E\{|x_{k,2}{|}^{2}\}=\frac{p_{\pi (k)}E_{\pi (k)}\Delta^{2}}{\beta_{k}}\leq P_{k}$, where

(12) $$\begin{array}{c} E_{k}=\frac{E\{|s_{k}{|}^{2}\}}{\Delta^{2}}. \end{array}$$

The power constraints can, thus, be expressed as follows:

(13) $$\begin{array}{c} \frac{1}{P_{\pi^{-1}\left(k\right)}\beta_{\pi^{-1}\left(k\right)}}\leq p_{k}\leq \frac{P_{\pi^{-1}\left(k\right)}\beta_{\pi^{-1}\left(k\right)}}{E_{k}\Delta^{2}},\;k=1,\ldots ,K. \end{array}$$

Our design can now be formulated into the following optimization problem.

**Problem** **1.** *Find the optimal power control vector p and permutation π under individual average power constraints:*

(14) $$\begin{array}{cc} \; & \max_{\left\{\pi ,p\right\}}\;\min_{\left\{s,\;\tilde{s}:\;s\neq \tilde{s}\right\}}\;f_{1}\left(p,s,\tilde{s}\right)+f_{2}\left(p,s,\tilde{s}\right)\\ \; & \;s.t.\;\frac{1}{P_{\pi^{-1}\left(k\right)}\beta_{\pi^{-1}\left(k\right)}}\leq p_{k}\leq \frac{P_{\pi^{-1}\left(k\right)}\beta_{\pi^{-1}\left(k\right)}}{E_{k}\Delta^{2}},\;k=1,\ldots ,K. \end{array}$$

For Problem 1, we first can attain that $f_{1}\left(p,s,\tilde{s}\right)\geq 0$ by applying the fundamental inequality in information theory ([43] Lemma 2.29), where the equality $f_{1}\left(p,s,\tilde{s}\right)=0$ holds if and only if

(15) $$\begin{array}{c} \left(\sum_{k=1}^{K}\frac{1}{p_{k}}+\sigma^{2}\right)\left(\sum_{k=1}^{K}p_{k}\left(\right|s_{k}{|}^{2}-|{\tilde{s}}_{k}{|}^{2})\right)-\left(|\sum_{k=1}^{K}s_{k}{|}^{2}-|\sum_{k=1}^{K}{\tilde{s}}_{k}{|}^{2}\right)=0. \end{array}$$

Considering the fact that the joint minimization of $f_{1}\left(p,s,\tilde{s}\right)$ and $f_{2}\left(p,s,\tilde{s}\right)$ over $\left\{s,\tilde{s}:s\neq \tilde{s}\right\}$ could be extremely tedious, we consider the minimization of $f_{2}\left(p,s,\tilde{s}\right)$ first, which is a lower bound of $D_{\mathrm{KL}}(X_{2}\left|\right|{\tilde{X}}_{2})$ as $f_{1}\left(p,s,\tilde{s}\right)\geq 0$. We will verify the condition when the minimum of $f_{1}\left(p,s,\tilde{s}\right)$ and $f_{2}\left(p,s,\tilde{s}\right)$ can be achieved simultaneously. Mathematically, we temporarily focus on solving the following optimization problem:

**Problem** **2.** *We find the power control coefficients p and permutation policy π, such that*

(16) $$\begin{array}{cc} \; & \max_{\left\{\pi ,p\right\}}\;\min_{\left\{s,\tilde{s}:\;s\neq \tilde{s}\right\}}\;f_{2}\left(p,s,\tilde{s}\right)=\frac{|\sum_{k=1}^{K}s_{k}-\sum_{k=1}^{K}{\tilde{s}}_{k}{|}^{2}}{\left(\sum_{k=1}^{K}\frac{1}{p_{k}}+\sigma^{2}\right)(\sum_{k=1}^{K}p_{k}|{\tilde{s}}_{k}{|}^{2}+\sigma^{2})-|\sum_{k=1}^{K}{\tilde{s}}_{k}{|}^{2}} \end{array}$$

(17) $$\begin{array}{cc} \; & \;s.t.\;\frac{1}{P_{\pi^{-1}\left(k\right)}\beta_{\pi^{-1}\left(k\right)}}\leq p_{k}\leq \frac{P_{\pi^{-1}\left(k\right)}\beta_{\pi^{-1}\left(k\right)}}{E_{k}\Delta^{2}},\;k=1,\ldots ,K. \end{array}$$

We first consider the inner optimization problem in Problem 2. The denominator of (16) is independent of s and the numerator is minimized when the sum terms $\sum_{k=1}^{K}s_{k}$ and $\sum_{k=1}^{K}{\tilde{s}}_{k}$ are the neighboring points on the sum constellation, where the minimum value of $|\sum_{k=1}^{K}s_{k}-\sum_{k=1}^{K}{\tilde{s}}_{k}{|}^{2}$ is $\Delta^{2}$. For notation simplicity, we define $\tilde{s}=\left(\tilde{v}+j\tilde{w}\right)\Delta$, where $\tilde{v}={\left[{\tilde{v}}_{1},\ldots ,{\tilde{v}}_{K}\right]}^{T}$ and $\tilde{w}={\left[{\tilde{w}}_{1},\ldots ,{\tilde{w}}_{K}\right]}^{T}$. As the power constraint given in (17) is independent of v and w, Problem 2 can be split into two subproblems as follows:

(18) $$\begin{array}{cc} \; & \max_{\tilde{v}}\;f_{3}\left(\tilde{v}\right)=\left(\sum_{k=1}^{K}\frac{1}{p_{k}}+\sigma^{2}\right)\left(\sum_{k=1}^{K}p_{k}{\tilde{v}}_{k}^{2}+\frac{\sigma^{2}}{\Delta^{2}}\right)-{\left(\sum_{k=1}^{K}{\tilde{v}}_{k}\right)}^{2}\\ \; & \;s.t.\;{\tilde{v}}_{k}\in {\left\{\pm \left(m-\frac{1}{2}\right)\times 2^{\sum_{\ell =1}^{k-1}N_{I,\ell }}\right\}}_{m=1}^{2^{N_{I,k}-1}},\;k=1,\ldots ,K. \end{array}$$

and

(19) $$\begin{array}{cc} \; & \max_{\tilde{w}}\;f_{4}\left(\tilde{w}\right)=\left(\sum_{k=1}^{K}\frac{1}{p_{k}}+\sigma^{2}\right)\left(\sum_{k=1}^{K}p_{k}{\tilde{w}}_{k}^{2}+\frac{\sigma^{2}}{\Delta^{2}}\right)-{\left(\sum_{k=1}^{K}{\tilde{w}}_{k}\right)}^{2}\\ \; & \;s.t.\;{\tilde{w}}_{k}\in {\left\{\pm \left(m-\frac{1}{2}\right)\times 2^{\sum_{\ell =1}^{k-1}N_{Q,\ell }}\right\}}_{m=1}^{2^{N_{Q,k}-1}},\;k=1,\ldots ,K. \end{array}$$

In the following, we only present the maximization of $f_{3}\left(\tilde{v}\right)$ over $\tilde{v}$ in (18), and the maximization of $f_{4}\left(\tilde{w}\right)$ over $\tilde{w}$ given in (19) follows similarly and, hence, is omitted for brevity. We now rewrite the objective function in (18) as

(20) $$\begin{array}{cc} f_{3}\left(\tilde{v}\right)= & \left[\frac{1}{p_{1}}+\left(\sum_{k=2}^{K}\frac{1}{p_{k}}+\sigma^{2}\right)\right]\left[p_{1}{\tilde{v}}_{1}^{2}+\left(\sum_{k=2}^{K}p_{k}{\tilde{v}}_{k}^{2}+\frac{\sigma^{2}}{\Delta^{2}}\right)\right]-{\left({\tilde{v}}_{1}+\sum_{k=2}^{K}{\tilde{v}}_{k}\right)}^{2}\\ = & \frac{1}{p_{1}}\left(\sum_{k=2}^{K}p_{k}{\tilde{v}}_{k}^{2}+\frac{\sigma^{2}}{\Delta^{2}}\right)+p_{1}{\tilde{v}}_{1}^{2}\left(\sum_{k=2}^{K}\frac{1}{p_{k}}+\sigma^{2}\right)+\left(\sum_{k=2}^{K}\frac{1}{p_{k}}+\sigma^{2}\right)\left(\sum_{k=2}^{K}p_{k}{\tilde{v}}_{k}^{2}+\frac{\sigma^{2}}{\Delta^{2}}\right)\\ \; & -2{\tilde{v}}_{1}\left(\sum_{k=2}^{K}{\tilde{v}}_{k}\right)-{\left(\sum_{k=2}^{K}{\tilde{v}}_{k}\right)}^{2}\\ = & \frac{1}{p_{1}}\left(\sum_{k=2}^{K}p_{k}{\tilde{v}}_{k}^{2}+\frac{\sigma^{2}}{\Delta^{2}}\right)+\frac{1}{p_{2}}\left(\sum_{k=3}^{K}p_{k}{\tilde{v}}_{k}^{2}+\frac{\sigma^{2}}{\Delta^{2}}\right)+p_{1}{\tilde{v}}_{1}^{2}\left(\sum_{k=2}^{K}\frac{1}{p_{k}}+\sigma^{2}\right)+p_{2}{\tilde{v}}_{2}^{2}\left(\sum_{k=3}^{K}\frac{1}{p_{k}}+\sigma^{2}\right)\\ \; & +\left(\sum_{k=3}^{K}\frac{1}{p_{k}}+\sigma^{2}\right)\left(\sum_{k=3}^{K}p_{k}{\tilde{v}}_{k}^{2}+\frac{\sigma^{2}}{\Delta^{2}}\right)-{\left(\sum_{k=3}^{K}{\tilde{v}}_{k}\right)}^{2}-2{\tilde{v}}_{1}\left(\sum_{k=2}^{K}{\tilde{v}}_{k}\right)-2{\tilde{v}}_{2}\left(\sum_{k=3}^{K}{\tilde{v}}_{k}\right)\\ = & \sum_{\ell =1}^{K-1}\frac{1}{p_{\ell }}\left(\sum_{k=\ell +1}^{K}p_{k}{\tilde{v}}_{k}^{2}+\frac{\sigma^{2}}{\Delta^{2}}\right)+\sum_{\ell =1}^{K-1}p_{\ell }{\tilde{v}}_{\ell }^{2}\left(\sum_{k=\ell +1}^{K}\frac{1}{p_{k}}+\sigma^{2}\right)+p_{K}{\tilde{v}}_{K}^{2}\sigma^{2}\\ \; & +\frac{\sigma^{4}}{\Delta^{2}}+p_{K}{\tilde{v}}_{K}^{2}\sigma^{2}+\frac{\sigma^{4}}{\Delta^{2}}-2\sum_{\ell =1}^{K-1}{\tilde{v}}_{\ell }\sum_{k=\ell +1}^{K}{\tilde{v}}_{k}\\ = & f_{5}\left(\tilde{v}\right)-f_{6}\left(\tilde{v}\right), \end{array}$$

where $f_{5}\left(\tilde{v}\right)=\sum_{\ell =1}^{K-1}\frac{1}{p_{\ell }}\left(\sum_{k=\ell +1}^{K}p_{k}{\tilde{v}}_{k}^{2}+\frac{\sigma^{2}}{\Delta^{2}}\right)+\sum_{\ell =1}^{K-1}p_{\ell }{\tilde{v}}_{\ell }^{2}\left(\sum_{k=\ell +1}^{K}\frac{1}{p_{k}}+\sigma^{2}\right)+p_{K}{\tilde{v}}_{K}^{2}\sigma^{2}+\frac{\sigma^{4}}{\Delta^{2}}+p_{K}{\tilde{v}}_{K}^{2}\sigma^{2}+\frac{\sigma^{4}}{\Delta^{2}}$, and $f_{6}\left(\tilde{v}\right)=2\sum_{\ell =1}^{K-1}{\tilde{v}}_{\ell }\sum_{k=\ell +1}^{K}{\tilde{v}}_{k}$.

We then can maximize $f_{5}\left(\tilde{v}\right)-f_{6}\left(\tilde{v}\right)$. In what follows, we will show that the maximization of $f_{5}\left(\tilde{v}\right)$ and the minimization of $f_{6}\left(\tilde{v}\right)$ can be achieved simultaneously. First, we can observe that the maximization of $f_{5}\left(\tilde{v}\right)$ is achieved when $|{\tilde{v}}_{k}|$, k=1,…,K, are maximized for a signal transmitted from every user.

We next consider the minimization of $f_{6}\left(\tilde{v}\right)$. To that end, we have,

(21) $$\begin{array}{c} \frac{\partial f_{6}\left(\tilde{v}\right)}{\partial {\tilde{v}}_{k}}=2\sum_{\ell =1,\ell \neq k}^{K}{\tilde{v}}_{\ell },k=1,\ldots ,K. \end{array}$$

The optimal value can be attained by enumeration of ${\tilde{v}}_{K}\in {\left\{\left(m-\frac{1}{2}\right)\times 2^{\sum_{\ell =1}^{K-1}N_{I,\ell }}\right\}}_{m=1}^{2^{N_{I,K}-1}}$, and ${\tilde{v}}_{K}\in {\left\{-\left(m-\frac{1}{2}\right)\times 2^{\sum_{\ell =1}^{K-1}N_{I,\ell }}\right\}}_{m=1}^{2^{N_{I,K}-1}}$.

1. If ${\tilde{v}}_{K}\in {\left\{\left(m-\frac{1}{2}\right)\times 2^{\sum_{\ell =1}^{K-1}N_{I,\ell }}\right\}}_{m=1}^{2^{N_{I,K}-1}}$, then for any ${\tilde{v}}_{k}\in {\left\{\pm \left(m-\frac{1}{2}\right)\times 2^{\sum_{\ell =1}^{k-1}N_{I,\ell }}\right\}}_{m=1}^{2^{N_{I,k}-1}}$, k=1,…,K−1, we have
  $$\begin{array}{cc} \; & \frac{\partial f_{6}\left(\tilde{v}\right)}{\partial {\tilde{v}}_{k}}=2{\tilde{v}}_{K}+2\sum_{\ell =1,\ell \neq k}^{K-1}{\tilde{v}}_{\ell }\geq 2\min{\tilde{v}}_{K}+2\min_{{\left\{{\tilde{v}}_{\ell }\right\}}_{\ell =1}^{K-1}}\sum_{\ell =1,\ell \neq k}^{K-1}{\tilde{v}}_{\ell }\\ \; & >2^{\sum_{\ell =1}^{K-1}N_{I,\ell }}+2\min_{{\left\{{\tilde{v}}_{\ell }\right\}}_{\ell =1}^{K-1}}\sum_{\ell =1}^{K-1}{\tilde{v}}_{\ell }=2^{\sum_{\ell =1}^{K-1}N_{I,\ell }}-2\left(2^{\sum_{\ell =1}^{K-1}N_{I,\ell }-1}-\frac{1}{2}\right)=1. \end{array}$$
  In this case, the optimal value of ${\left\{{\tilde{v}}_{k}\right\}}_{k=1}^{K-1}$ to minimize $f_{6}\left(\tilde{v}\right)$ is given by
  $$\begin{array}{c} {\tilde{v}}_{k}=-\left(2^{N_{I,k}}-1\right)\times 2^{\sum_{\ell =1}^{k-1}N_{I,\ell }-1},\;\mathrm{for}\;k=1,\ldots ,K-1. \end{array}$$
  Note that $\frac{\partial f_{6}\left(\tilde{v}\right)}{\partial {\tilde{v}}_{K}}=2\sum_{\ell =1}^{K-1}{\tilde{v}}_{\ell }<0$, then for ${\tilde{v}}_{K}\in {\left\{\left(m-\frac{1}{2}\right)\times 2^{\sum_{\ell =1}^{K-1}N_{\ell }}\right\}}_{m=1}^{2^{N_{I,K}-1}}$, the optimal value of ${\tilde{v}}_{K}$ is ${\tilde{v}}_{K}=\left(2^{N_{K}}-1\right)\times 2^{\sum_{\ell =1}^{K-1}N_{\ell }-1}$.
2. If ${\tilde{v}}_{K}\in {\left\{-\left(m-\frac{1}{2}\right)\times 2^{\sum_{\ell =1}^{K-1}N_{\ell }}\right\}}_{m=1}^{2^{N_{I,K}-1}}$, for ${\tilde{v}}_{k}\in {\left\{\pm \left(m-\frac{1}{2}\right)\times 2^{\sum_{\ell =1}^{k-1}N_{\ell }}\right\}}_{m=1}^{2^{N_{I,k}-1}}$, k=1,…,K−1, we have
  $$\begin{array}{cc} \; & \frac{\partial f_{6}\left(\tilde{v}\right)}{\partial {\tilde{v}}_{k}}=2{\tilde{v}}_{K}+2\sum_{\ell =1,\ell \neq k}^{K-1}{\tilde{v}}_{\ell }\leq 2{\tilde{v}}_{K}+2\max_{{\left\{{\tilde{v}}_{\ell }\right\}}_{\ell =1}^{K-1}}\sum_{\ell =1,\ell \neq k}^{K}{\tilde{v}}_{\ell }\\ \; & <-2^{\sum_{\ell =1}^{K-1}N_{I,\ell }}+2\max_{{\left\{{\tilde{v}}_{\ell }\right\}}_{\ell =1}^{K-1}}\sum_{\ell =1}^{K-1}{\tilde{v}}_{\ell }=-2^{\sum_{\ell =1}^{K-1}N_{I,\ell }}+2\left(2^{\sum_{\ell =1}^{K-1}N_{I,\ell }-1}-\frac{1}{2}\right)=-1. \end{array}$$
  In this case, the optimal value of ${\left\{{\tilde{v}}_{k}\right\}}_{k=1}^{K-1}$ to minimize $f_{6}\left(\tilde{v}\right)$ is given by
  $$\begin{array}{c} {\tilde{v}}_{k}=\left(2^{N_{k}}-\frac{1}{2}\right)\times 2^{\sum_{\ell =1}^{k-1}N_{I,\ell }},\;\mathrm{for}\;k=1,\ldots ,K-1. \end{array}$$
  In addition, we note that $\frac{\partial f_{6}\left(\tilde{v}\right)}{\partial {\tilde{v}}_{K}}=2\sum_{\ell =1}^{K-1}{\tilde{v}}_{\ell }<0$, then for ${\tilde{v}}_{K}\in {\left\{-\left(m-\frac{1}{2}\right)\times 2^{\sum_{\ell =1}^{K-1}N_{\ell }}\right\}}_{m=1}^{2^{N_{I,K}-1}}$, the optimal value of ${\tilde{v}}_{K}$ is ${\tilde{v}}_{K}=-\left(2^{N_{I,K}}-1\right)\times 2^{\sum_{\ell =1}^{K-1}N_{I,\ell }-1}$.

In summary, the maximum value of $f_{6}\left(\tilde{v}\right)=2\sum_{\ell =1}^{K-1}{\tilde{v}}_{\ell }\sum_{k=\ell +1}^{K}{\tilde{v}}_{k}$ can be achieved by ${\tilde{v}}^{⋆}={\left[{\tilde{v}}_{1}^{⋆},\ldots ,{\tilde{v}}_{K}^{⋆}\right]}^{T}$ where

(22) $$\begin{array}{c} {\tilde{v}}_{k}^{⋆}=\left\{\begin{array}{cc} -\left(2^{N_{I,k}-1}-\frac{1}{2}\right)\times 2^{\sum_{\ell =1}^{k-1}N_{I,\ell }}, & \mathrm{for}\;k=1,\ldots ,K-1;\\ \left(2^{N_{I,K}-1}-\frac{1}{2}\right)\times 2^{\sum_{\ell =1}^{K-1}N_{I,\ell }}, & \mathrm{for}\;k=K, \end{array}\right. \end{array}$$

or equivalently

(23) $$\begin{array}{c} {\tilde{v}}_{k}^{⋆}=\left\{\begin{array}{cc} \left(2^{N_{I,k}-1}-\frac{1}{2}\right)\times 2^{\sum_{\ell =1}^{k-1}N_{I,\ell }}, & \mathrm{for}\;k=1,\ldots ,K-1;\\ -\left(2^{N_{I,K}-1}-\frac{1}{2}\right)\times 2^{\sum_{\ell =1}^{K-1}N_{I,\ell }}, & \mathrm{for}\;k=K. \end{array}\right. \end{array}$$

For both cases, we can observe that $f_{5}\left(\tilde{v}\right)$ as defined in (21) is also maximized by ${\tilde{v}}^{⋆}$ as $|{\tilde{v}}_{k}|$, k=1,…,K, are maximized. Due to the symmetry of the solutions given in (22) and (23), in what follows, we only consider the solution given in (22). In this case, the sum constellation for achieving the inner minimum is

(24) $$\begin{array}{cc} \; & \sum_{k=1}^{K}{\tilde{s}}_{k}=\sum_{k=1}^{K}{\tilde{v}}_{k}+j{\tilde{w}}_{k}\\ \; & =\left[\frac{1+j}{2}+\left(2^{N_{I,K}-1}-1\right)\times 2^{\sum_{\ell =1}^{K-1}N_{I,\ell }}+j\left(2^{N_{Q,K}-1}-1\right)\times 2^{\sum_{\ell =1}^{K-1}N_{Q,\ell }}\right]\Delta . \end{array}$$

We now have the following remark:

**Remark** **1.** *When $N_{I,K}=N_{Q,K}=1$, the solution given in* (22)*, which minimizes $f_{2}\left(p,s,\tilde{s}\right)$ also minimizes $f_{1}\left(p,s,\tilde{s}\right)$.*

**Proof.**  For the solution of $\tilde{s}$ given in (22), the sum constellation is given in (24). When $N_{I,K}=N_{Q,K}=1$, we have $\sum_{k=1}^{K}{\tilde{s}}_{k}=\frac{1+j}{2}\Delta$, and we can let $\sum_{k=1}^{K}s_{k}=\frac{1-j}{2}\Delta$. Inserting them back into (15), we have $f_{1}\left(p,s,\tilde{s}\right)=0$. That is, the values that minimize $f_{2}\left(p,s,\tilde{s}\right)$ also minimizes $f_{1}\left(p,s,\tilde{s}\right)$. This completes the proof.    □

We now consider the outer optimization problem, where the objective function is a monotonically decreasing function against the term $f_{7}\left(\pi ,p\right)=\left(\sum_{k=1}^{K}\frac{1}{p_{k}}+\sigma^{2}\right)\left(\sum_{k=1}^{K}p_{k}E_{k}+\frac{\sigma^{2}}{\Delta^{2}}\right)$. The optimization problem can be reformulated as

(25) $$\begin{array}{cc} \; & \min_{\pi ,p}\;f_{7}\left(\pi ,p\right)=\left(\sum_{k=1}^{K}\frac{1}{p_{k}}+\sigma^{2}\right)\left(\sum_{k=1}^{K}p_{k}E_{k}+\frac{\sigma^{2}}{\Delta^{2}}\right)\\ \; & \;s.t.\;\frac{1}{p_{k}}\leq P_{\pi^{-1}\left(k\right)}\beta_{\pi^{-1}\left(k\right)},\;p_{k}E_{k}\Delta^{2}\leq P_{\pi^{-1}\left(k\right)}\beta_{\pi^{-1}\left(k\right)},\;k=1,\ldots ,K. \end{array}$$

The optimization problem in (25) can be resolved by first fixing π to find the optimal value of p, and then performing further optimization on π. To that end, we can observe from (25) that, for any given π, the feasible range of $\Delta^{2}$ is given by $\Delta^{2}\leq \frac{P_{\pi^{-1}\left(k\right)}\beta_{\pi^{-1}\left(k\right)}}{p_{k}E_{k}}\leq \frac{P_{\pi^{-1}\left(k\right)}^{2}\beta_{\pi^{-1}\left(k\right)}^{2}}{E_{k}}$ for k=1,…,K, or equivalently $\Delta^{2}\leq \min\;{\left\{\frac{P_{\pi^{-1}\left(k\right)}^{2}\beta_{\pi^{-1}\left(k\right)}^{2}}{E_{k}}\right\}}_{k=1}^{K}$. By the Cauchy–Schwarz inequality, we have

$$\begin{array}{cc} \; & f_{7}\left(\pi ,p\right)=\left(\sum_{k=1}^{K}\frac{1}{p_{k}}+\sigma^{2}\right)\left(\sum_{k=1}^{K}p_{k}E_{k}+\frac{\sigma^{2}}{\Delta^{2}}\right)\\ \; & \overset{\left(a\right)}{\geq }{\left(\sum_{k=1}^{K}\frac{1}{\sqrt{p_{k}}}\sqrt{p_{k}E_{k}\Delta^{2}}+\frac{\sigma^{2}}{\Delta }\right)}^{2}=\underset{f_{8}\left(\pi \right)}{{\left(\underset{︸}{\sum_{k=1}^{K}\sqrt{E_{k}}+\frac{\sigma^{2}}{\Delta }}\right)}^{2}}, \end{array}$$

where the inequality in (a) holds if and only if $\frac{\sqrt{p_{k}E_{k}}}{1/\sqrt{p_{k}}}=\frac{1}{\Delta }$, for k=1,…K. Or equivalently, the optimal power allocation is $p={\left[p_{1}^{⋆},\ldots ,p^{⋆}\right]}^{T}$, where $p_{k}^{⋆}=\frac{1}{\sqrt{E_{k}}\Delta }$ for k=1,…,K. Our next task is to check if the power constraint on $p_{k}^{⋆}$ given in (25) is violated or not. For $\Delta^{2}\leq \min\;{\left\{\frac{P_{\pi^{-1}\left(k\right)}^{2}\beta_{\pi^{-1}\left(k\right)}^{2}}{E_{k}}\right\}}_{k=1}^{K}$, we have

$$\begin{array}{cc} \; & \frac{1}{p_{k}^{⋆}}=\sqrt{E_{k}}\Delta \leq P_{\pi^{-1}\left(k\right)}\beta_{\pi^{-1}\left(k\right)},\\ \; & p_{k}^{⋆}E_{k}\Delta^{2}=\sqrt{E_{k}}\Delta \leq P_{\pi^{-1}\left(k\right)}\beta_{\pi^{-1}\left(k\right)},\;\mathrm{for}\;k=1,\ldots ,K, \end{array}$$

where no power constraints are violated for p. Finally, the optimization problem on π can be given by

$$\begin{array}{cc} \; & \min_{\pi }\;f_{8}\left(\pi \right)=\sum_{k=1}^{K}\sqrt{E_{k}}+\frac{\sigma^{2}}{\Delta }\\ \; & s.t.\;\Delta^{2}\leq \frac{P_{\pi^{-1}\left(k\right)}^{2}\beta_{\pi^{-1}\left(k\right)}^{2}}{E_{k}},\;k=1,\ldots ,K. \end{array}$$

Or equivalently, we aim to solve

(26) $$\begin{array}{cc} \; & \max_{\pi }\;\Delta \;s.t.\;\Delta^{2}\leq \frac{P_{k}^{2}\beta_{k}^{2}}{E_{\pi (k)}},\;k=1,\ldots ,K. \end{array}$$

Before proceeding, we establish the following lemma.

**Lemma** **2.** *Suppose that two positive sequences ${\left\{a_{n}\right\}}_{n=1}^{N}$ and ${\left\{b_{n}\right\}}_{n=1}^{N}$ are arranged both in a nondecreasing order. If we let *Π* denote the set containing all the possible permutations of 1,2,⋯,N, then, the solution to the optimization problem, $\max_{\pi \in \Pi }\min{\left\{\frac{a_{k}}{b_{\pi (k)}}\right\}}_{k=1}^{K}$, is given by $\pi^{⋆}\left(k\right)=k$ for k=1,2,⋯,K.*

By Lemma 2, and note that $P_{1}\beta_{1}\leq \ldots \leq P_{k}\beta_{K}$, to maximize Δ, we should let $E_{\pi (1)}\leq \ldots \leq E_{\pi (K)}$, i.e., the average power of the sub-constellations should be arranged in ascending order. All the above discussions can be summarized into the following theorem:

**Theorem** **1.** *The users are ordered, such that $P_{1}\beta_{1}\leq P_{2}\beta_{2}\leq \ldots \leq P_{k}\beta_{K}$, and we define $\Delta^{⋆}=\min_{k}{\left\{\frac{P_{k}\beta_{k}}{\sqrt{E_{k}}}\right\}}_{k=1}^{K}$, the optimal transmit power for all users can be given by $p^{⋆}={\left[\frac{1}{\sqrt{E_{1}}\Delta^{⋆}},\ldots ,\frac{1}{\sqrt{E_{K}}\Delta^{⋆}}\right]}^{T}$. In addition, the optimal permutation matrix is the identity matrix, i.e., $\Pi =I_{K}$.*

The above procedure can be summarized in Algorithm 1.
**Algorithm 1** UF-MUSTM for multi-user massive SIMO systems.1: Given the large-scale fading diagonal matrix $D=\mathrm{diag}\{\beta_{1},\ldots ,\beta_{K}\}$, power constraint $P_{k}$, constellation size of each user $N_{k}=N_{I,k}+N_{Q,k}$, k=1,…,K.2: Obtain the optimal permutation matrix Π (or equivalently permutation π) as defined in (9), the optimal value of Δ, and the power allocation vector of $p=[p_{1},\ldots ,p_{K}]$ by Theorem 1, where $E_{k}$ is defined in (12).3: For given user constellation size $N_{k}=N_{I,k}+N_{Q,k}$, k=1,…,K and Δ, obtain the UDCG $Q={⊎}_{k=1}^{K}X_{k}$ by Lemma 1.4: Construct the transmitted signal vector $X_{2}=\left[x_{1},x_{2}\right]=D^{-1/2}\Pi S_{2}$, in which $S_{2}=\left[s_{1},s_{2}\right]$ with $s_{1}={\left[\frac{1}{\sqrt{p_{1}}},\ldots ,\frac{1}{\sqrt{p_{K}}}\right]}^{T}$, and $s_{2}={\left[\sqrt{p_{1}}s_{1},\ldots ,\sqrt{p_{K}}s_{K}\right]}^{T}$, as defined in (8) such that $s_{k}\in X_{k}$, k=1,…,K.5: For each transmission, the transmitted signal $X_{2}$ can be recovered by solving (11) (or equivalently (6)).

We have the following remark on Algorithm 1.

**Remark** **2.** *Computational complexity and training overhead analysis.*

- *The main complexity of our algorithm for signal recovery comes from solving* (11) *in Step 5. More specifically, the complexity of evaluating $∥y_{1}∥$, $∥y_{2}∥$, and $y_{2}^{H}y_{1}$ in* (11) *is O(M). For each transmitted signal matrix, the complexity of evaluating all possible $b=\sum_{k=1}^{K}p_{k}{\left|s_{k}\right|}^{2}+\sigma^{2}$, and $c=\sum_{k=1}^{K}s_{k}$ in* (11) *is $O(2^{N})$, where $2^{N}$ is the size of the sum constellation $Q={⊎}_{k=1}^{K}X_{k}$. In conclusion, for each transmitted signal estimation, the overall complexity is $O\left(M\right)+O\left(2^{N}\right)$, which is similar to other noncoherent designs [30,31,44]. In contrast, for the linear receiver, such as zero-forcing (ZF), the complexity is $O\left(K^{2}M\right)+O\left(K^{3}\right)$ [20,45].*
- *Our design is based on a noncoherent ML receiver, which only needs the estimation of the second-order channel statistics D, which can be done when the system is idle, such as the schemes in [30,31,44]; hence, there is no overhead for instantaneous CSI estimation. On the other hand, the instantaneous channel estimation, including the classical least-square (LS) and minimum mean-square error (MMSE) estimators [46], needs to send pilot symbols with a length proportional to the number of users K in each channel coherence time $T_{c}\geq K$ [20].*

## 4. Simulation Results and Discussions

In this section, computer simulations are performed to demonstrate the superior performance of our design in comparison with existing benchmarks. In our simulations, the small-scale fading is assumed to be the normalized Rayleigh fading. The path-loss as a function of the transmission distance *d* at the antenna’s far-field can be approximated by

$$\begin{array}{c} 10\log_{10}L=20\log_{10}\left(\frac{\lambda }{4\pi d_{0}}\right)-10\gamma \log_{10}\left(\frac{d}{d_{0}}\right)-\psi ,\;d\geq d_{0}, \end{array}$$

where $d_{0}=100$ m is the reference distance, $\lambda =v_{c}/f_{c}$ ($f_{c}=3$ GHz) is the wavelength of carrier, γ=3.71 is the path-loss exponent [47]. In the above model, $\psi ∼N(0,\sigma_{\psi }^{2})$ ($\sigma_{\psi }=3.16$) is the Gaussian random shadowing attenuation resulting from the blockage of objects. For the receiver, we assume that the noise power is $10\log_{10}\sigma^{2}=10\log_{10}N_{0}B_{w}=10\log_{10}3.2\times 10^{-10}=-125.97\;\mathrm{dB}$ where the channel bandwidth $B_{w}=20$ MHz, and $N_{0}=k_{0}T_{0}10^{F_{0}/10}$ is the power spectral density of noise with $k_{0}=1.38\times 10^{-23}$ J/K being the Boltzmann constant, reference temperature $T_{0}=290$ K (“room temperature”), and noise figure $F_{0}=6$ dB. For clarity, all the simulation parameters are summarized in Table 1.

**Figure 1:**
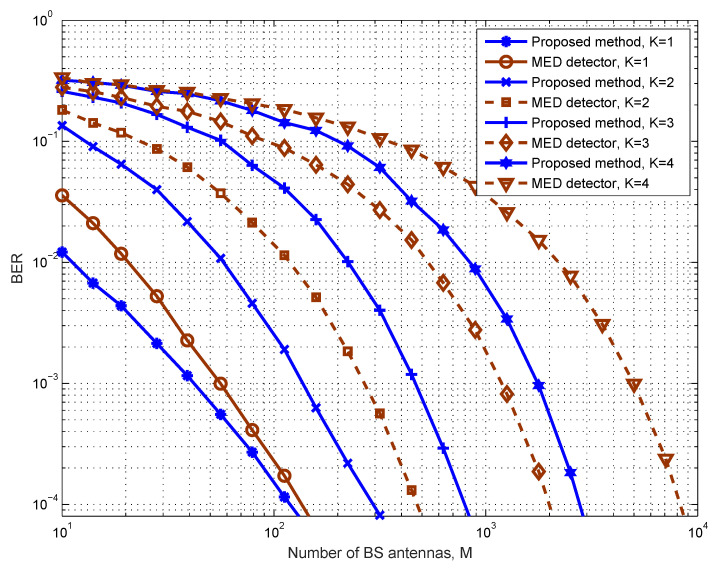
Comparison of the proposed scheme with the MED detector on the average BER of all users versus *M*, 4-QAM is used by all users with an average power constraint.

We first examine the error performance of the proposed design under the instantaneous average power constraint for different user numbers, as illustrated in Figure 1. It is assumed that the average power upper bound is $P_{k}=316$ mW (25 dBm), ∀k. All the *K* users are assumed to be uniformly distributed within the cell of radius *d*. It can be observed that, as the number of users increases, the error performance deteriorates quickly, which is caused by the mutual interference among users. Then, more BS antennas are needed to achieve the same average BER. We also compare our design with the max–min Euclidean distance (MED)-based method proposed in [30,31]. Since we use two time slots, while the MED methods only need one time slot, we assume that 2-PAM constellations are adopted by all users for the MED-based design. We can see from the figure that the proposed approach significantly outperforms the MED-based method in terms of BER in all simulated cases.

**Figure 2:**
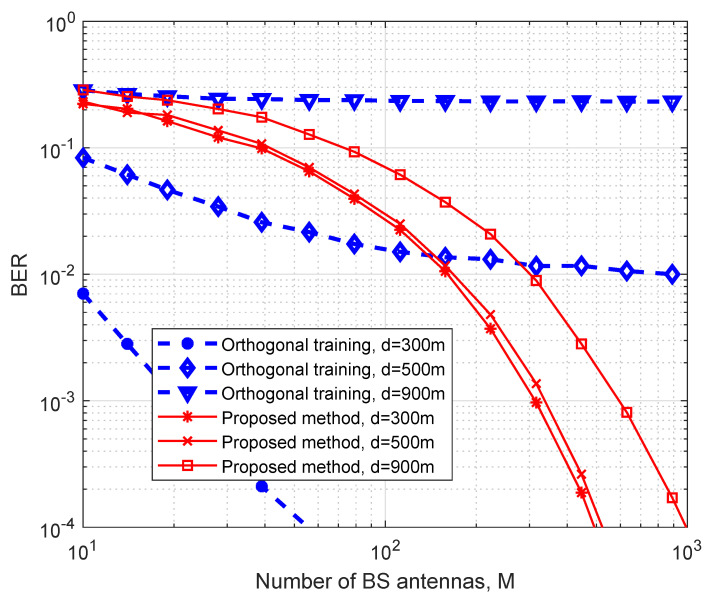
The comparison between the proposed and the orthogonal training method with K=3 users and 4 time slots.

We next compare the error performance of the proposed framework with the conventional ZF receiver using orthogonal training sequence. The results are shown in Figure 2. In this simulation, we consider a system setup with K=3 users. For the orthogonal training-based method, at least four time slots (three time slots for training and one time slot for data transmission) are needed, and we assume that the channel coefficients are quasi-static within these consecutive time slots. As 4-QAM is adopted by each user for the proposed scheme, 64-QAM is correspondingly adopted for the training-based approach in order to make a fair comparison. For the channel training algorithm, we consider that a widely used least-square (LS) channel estimator is employed [46]. It can be observed from Figure 2 that when the antenna number *M* is small and the channel gain is large (i.e., the distance *d* is small), the training-based method outperforms the proposed design in terms of BER. However, when the antenna number is relatively large, the proposed design has a better error performance, especially at the cell edge. The rationale is that without a reliable CSI, especially at low signal-to-noise ratio (SNR) regimes, coherent detection suffers from inferior decoding performance.

**Figure 3:**
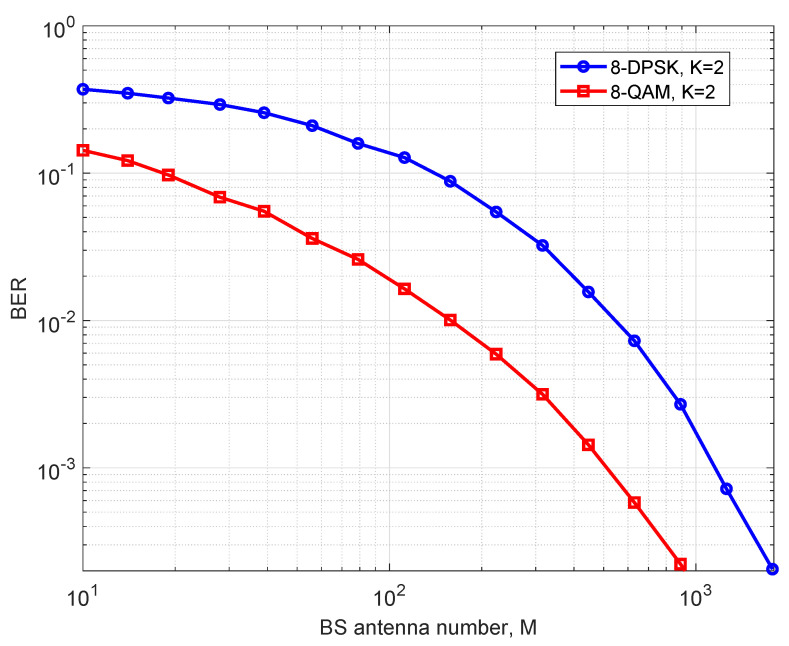
The comparison between the proposed and the noncoherent receiver with 8-QAM and 8-DPSK, respectively.

It is finally worth mentioning that a related noncoherent multi-user massive MIMO system was designed in [44] for differential phase shift keying (DPSK) constellations. All users’ transmitted information is modulated based on the phase offset between consecutive symbols. Indeed, the DBPSK and DQPSK constellations, which have an optimal scale between each sub-constellation, are specific instances of our QAMD. However, for larger constellations, such as 8-DPSK, our design has a greater normalized minimal Euclidean distance. The resulting sum constellation of two 8-DQPSKs is not a regular constellation anymore, just as studied in [48]. Also, in [44], the actual transmitted power of each user is not given explicitly and, hence, the optimal power allocation under both the average and the peak power constraint case is hard to evaluate. To make a comparison, especially when the constellation size is large, we compare the 8-DPSK constellation suggested in [44] with the optimal scale of 1.765 between the two sub-constellations with the rectangular 8-QAM constellation in our case. The error performance of [44] and our proposed design with two users, using 8-DPSK and 8-QAM respectively, is studied in Figure 3. It can be observed that our scheme with 8-QAM sub-constellation has a better error performance than [44] using 8-DPSK constellation, since the normalized minimal distance for our constellation is larger. Also, it should be pointed out that the resulting sum constellation in [44] is not a regular constellation, and it must be either computed or stored in advance. The detection of the sum constellation typically requires an exhaustive search over the whole constellation. In addition, the optimal power scale for general DPSK needs to be optimized by numerical methods. In contrast, our design leads to a regular QAM sum constellation. Furthermore, the optimal transmit powers of all users and the sub-constellation assignments among them have been provided in closed form.

## 5. Conclusions

In this paper, a non-orthogonal and noncoherent massive MIMO (nn-mMIMO) framework with the objective of enabling scalable URLLC applications was developed based on a new uniquely factorable multi-user space-time modulation (UF-MUSTM) scheme. For the MUSTM code design, a simple yet systematic construction method based on the concept of the QAM division was devised. Assuming that large-scale fading coefficients are known at the base station, the detailed transmission scheme and the corresponding noncoherent detector were carefully designed. We further optimized the proposed design framework by jointly optimizing the constellations of multiple users. Specifically, we implemented a max–min Kullback–Leibler (KL) divergence-based design criterion, where we jointly optimized the transmitted powers of all users and the sub-constellation assignments among them. Simulations demonstrated that the optimized nn-mMIMO framework has better reliability performance compared to the state-of-the-art benchmarking schemes.

## Figures and Tables

**Table 1:** Simulation parameters.

Parameter	Value
Cell radius $d_{\max}$	1000 m
Reference distance $d_{0}$	100 m
Carrier frequency $f_{c}$	3 GHz
Channel bandwidth $B_{w}$	20 MHz
Path loss exponent γ	3.71
Reference temperature/Noise figure	290 K/6 dB
Standard deviation of shadow fading $\sigma_{\psi }$	3.16

## Data Availability

Not applicable.

## Appendix A

### Appendix A.1. Proof of Proposition 1

**Proof.** We first show the sufficiency of Proposition 1 for the considered massive MIMO system with an unlimited number of antennas. By Assumption 1 on channel statistics and the central limit theory, we have $\lim_{M\to \infty }\frac{G^{H}G}{M}=I_{K}$ and $\lim_{M\to \infty }\frac{\Xi^{H}\Xi }{M}=\sigma^{2}I_{T}$. Now, the receiver can employ a simple correlation-based detector by calculating $R_{M}=\frac{Y^{H}Y}{M}$. When the antenna array size goes to infinity, we have $\lim_{M\to \infty }R_{M}-\sigma^{2}I_{T}=R$, where R is a T×T Hermitian-positive semidefinite matrix, such that $R=X_{T}^{H}DX_{T}$. Now, $X_{T}$ can be uniquely determined by an exhaustive search since for any $X_{T},{\tilde{X}}_{T}\in M_{K\times T}$ with $X_{T}^{H}DX_{T}={\tilde{X}}_{T}^{H}D{\tilde{X}}_{T}$, we have $X_{T}={\tilde{X}}_{T}$. Next, we show the necessity of Proposition 1. Suppose that there exist $X_{T},{\tilde{X}}_{T}\in M_{K\times T}$, such that $X_{T}\neq {\tilde{X}}_{T}$ for $X_{T}^{H}DX_{T}={\tilde{X}}_{T}^{H}D{\tilde{X}}_{T}$. As a consequence, $X_{T}$ and ${\tilde{X}}_{T}$ will have exactly the same likelihood function as shown in Equation (3); hence, they are indistinguishable by the ML detector, where the reliable recovery of the transmitted signals cannot be guaranteed. This completes the proof of Proposition 1.   □

### Appendix A.2. Proof of Proposition 2

**Proof.** Let $X_{2}=D^{-1/2}S_{2}$ and ${\tilde{X}}_{2}=D^{-1/2}{\tilde{S}}_{2}$. Then, if $X_{2}^{H}DX_{2}={\tilde{X}}_{2}^{H}D{\tilde{X}}_{2}$, and note that $\Pi^{H}\Pi =I_{M}$, we have $S_{2}^{H}S_{2}={\tilde{S}}_{2}^{H}{\tilde{S}}_{2}$. As a consequence, we have $\sum_{k=1}^{N}s_{k}=\sum_{k=1}^{K}{\tilde{s}}_{k}$, where $s_{k},{\tilde{s}}_{k}\in X_{k}$. Since ${\left\{X_{k}\right\}}_{k=1}^{K}$ form a UDCG, by Lemma 1, we can attain $s_{k}={\tilde{s}}_{k}$, or equivalently, $S_{2}={\tilde{S}}_{2}$, and now we have $X_{2}={\tilde{X}}_{2}$. This completes the proof of Proposition 2.   □

### Appendix A.3. Proof of Lemma 2

**Proof.** Let $m=\arg\min_{k=1,2,\ldots ,N}\left\{\frac{a_{k}}{b_{\pi^{*}\left(k\right)}}\right\}=\arg\min_{k}\left\{\frac{a_{k}}{b_{k}}\right\}$. In other words, *m* is the index, such that $q_{m}=\frac{a_{m}}{b_{m}}=\min_{k=1,2,\ldots ,N}\left\{\frac{a_{k}}{b_{k}}\right\}$. Now, we want to show that $q_{m}=\max_{(\pi (1),\pi (2),\ldots ,\pi (N))\in U}\min_{k=1,2,\cdots ,N}\left\{\frac{a_{k}}{b_{\pi (k)}}\right\}$. To that end, we divide U into two mutually exclusive subsets, i.e., P={(π(1),π(2),…,π(N))|π(m)≠m} and U\P={(π(1),π(2),…,π(N))|π(m)=m}. Consider the following cases:

- $(\pi^{\prime }\left(1\right),\pi^{\prime }\left(2\right),\ldots ,\pi^{\prime }\left(N\right))\in P$. In this case, there exists an ℓ≠m, such that $\pi^{\prime }\left(\ell \right)=m$ and, hence, $b_{\pi^{\prime }\left(\ell \right)}=b_{m}$. If ℓ<m, then, we have $\frac{a_{\ell }}{b_{\pi^{\prime }\left(\ell \right)}}=\frac{a_{\ell }}{b_{m}}\leq \frac{a_{m}}{b_{m}}=q_{m}$. If ℓ>m, there exists an n≤m, such that $\pi^{\prime }\left(n\right)>m$ by the property of permutation. Then, we have $\frac{a_{n}}{b_{\pi^{\prime }\left(n\right)}}\leq \frac{a_{m}}{b_{\pi^{\prime }\left(n\right)}}\leq \frac{a_{m}}{b_{m}}=q_{m}$. Therefore, we conclude $\min_{k=1,2,\ldots ,N}\left\{\frac{a_{k}}{b_{\pi^{\prime }\left(k\right)}}\right\}\leq q_{m}$ for any $(\pi^{\prime }\left(1\right),\pi^{\prime }\left(2\right),\ldots ,\pi^{\prime }\left(N\right))\in P$. Or equivalently, $\max_{(\pi (1),\pi (2),\ldots ,\pi (N))\in P}\min_{k=1,2,\cdots ,N}\left\{\frac{a_{k}}{b_{\pi (k)}}\right\}\leq q_{m}$.
- $(\pi^{\prime }\left(1\right),\pi^{\prime }\left(2\right),\ldots ,\pi^{\prime }\left(N\right))\in U\backslash P$. In this case, $\pi^{\prime }\left(m\right)=m$ and, hence, we have $\min_{k=1,2,\cdots ,N}\left\{\frac{a_{k}}{b_{\pi^{\prime }\left(k\right)}}\right\}\leq \frac{a_{m}}{b_{\pi^{\prime }\left(m\right)}}=\frac{a_{m}}{b_{m}}=q_{m}$. Therefore, $\max_{(\pi (1),\pi (2),\cdots ,\pi (N))\in U\backslash P}$
  $\min_{k=1,2,\cdots ,N}\left\{\frac{a_{k}}{b_{\pi (k)}}\right\}\leq q_{m}$.

In conclusion, we have $\max_{\pi \in U}\min_{k=1,2,\cdots ,N}\left\{\frac{a_{k}}{b_{\pi (k)}}\right\}\leq q_{m}$. In the following, we aim to prove that the equality is achievable for certain (π(1),π(2),…,π(K)). By setting $\left(\pi \left(1\right),\pi \left(2\right),\cdots ,\pi \left(N\right)\right)=\left(\pi^{*}\left(1\right),\pi^{*}\left(2\right),\ldots ,\pi^{*}\left(N\right)\right)$ and then, from the construction process above, we can find that for the given sequences $a_{1}\leq a_{2}\leq \cdots \leq a_{N}$ and $b_{1}\leq b_{2}\leq \cdots \leq b_{N}$, $\min_{k=1,2,\cdots ,N}\left\{\frac{a_{k}}{b_{\pi^{*}\left(k\right)}}\right\}=\frac{a_{m}}{b_{m}}=q_{m}$. Hence, the equality is achievable for $\left(\pi^{*}\left(1\right),\pi^{*}\left(2\right),\cdots ,\pi^{*}\left(N\right)\right)$. This completes the proof.   □
